# The Ring Fencing Mechanism: a case study of innovative self-financing approach for energy efficiency upgrades

**DOI:** 10.12688/openreseurope.14731.1

**Published:** 2022-05-05

**Authors:** Ruchi Agrawal, Luciano De Tommasi, Padraig Lyons

**Affiliations:** 1 International Energy Research Centre, University College Cork, Cork, T12R5CP, Ireland

**Keywords:** Energy Audit, Energy Management, Energy Efficiency, Energy Conservation Measures, Investments, Savings

## Abstract

This paper introduces an enhanced energy auditing process including a ring-fencing mechanism for the selection of energy efficiency measures considering a multi-stage planning of the energy retrofitting project. The proposed ring-fencing approach enables SMEs to overcome the barrier of lack of capital for the implementation of energy efficiency measures by implementing first no-cost measures and only after that energy savings are accumulated considering the installation of low-cost, medium-cost and finally high-cost measures. The advantages of the proposed methodology are illustrated by means of three case studies, where a variety of energy efficiency measures were first identified throughout the auditing process involving three different SMEs, then most effective measures were selected and scheduled to be implemented according to a multi-annual plan while considering budget and operational constraints. The results of the pilot studies show that the business owners have improved their decision-making with respect to energy efficiency upgrades by engaging in the auditing process and accepting the recommendations about the suggested interventions to maximize financial (and environmental) benefits.

## Introduction

Small and Medium Enterprises (SMEs) have enormous potential to save energy as they account for 99.98% of European enterprises ( Muller *et al.*, 2017) are responsible for approximately 13% of total energy demand ( IEA, 2017). SMEs of European Member States would play a vital role towards achieving combined 32.5% improvement in energy efficiency by 2030 as outlined in Energy Efficiency Directive targets. 

Studies have estimated that only 25% of SMEs in Europe have undertaken an energy audit ( CHANGE Project, 2010) to date, resulting in harnessing only a small energy saving potential of SMEs. The elimination of the costs associated with energy auditing and the availability of a professional auditor going on-site have been considered fundamental to increase the uptake of energy efficiency measures from SMEs ( Redmond & Walker, 2016). However, removing these barriers does not guarantee that the energy efficiency measures recommended by the auditors would be implemented by the business owner, since the costs involved may be unaffordable in most cases ( Redmond & Walker, 2016; Thollander *et al.*, 2015). Energy auditing programs for SMEs (such as the German one ( Fleiter *et al.*, 2012)) have not been effective in reducing financial barriers; this type of barrier can be overcome by means of soft loan programs, use of contracting or direct grants for the investment cost. This paper argues that the energy auditor should offer a solution to the decision maker for overcoming the barrier associated with the initial high investment costs of the energy efficiency upgrades which enable the self-financing of the energy retrofitting project using the energy savings accumulated with the initial implementation of no-cost and low-cost measures. The suggested approach requires a thorough energy audit to accurately identify all the energy efficiency measures applicable and their costs, as opposed to the so-called walk-through audit, which is characterized by a low level of detail and a short time of the auditor spent at the company. The walk-through audit is cheap (or even free) but results in a less-detailed report about the most cost-effective energy-efficiency upgrades which can be implemented by the SME, and in many cases fails in getting the full commitment of the decision maker in executing the energy-efficiency plan ( Palm & Backman, 2020).

Several studies have found the reason for this as SMEs’ various actual and perceived barriers to energy efficiency. These barriers include lack of time, resource, in-house expertise, finance and the low priority nature of energy efficiency compared to other business needs ( Fresner *et al.*, 2017).

**Figure 1.**
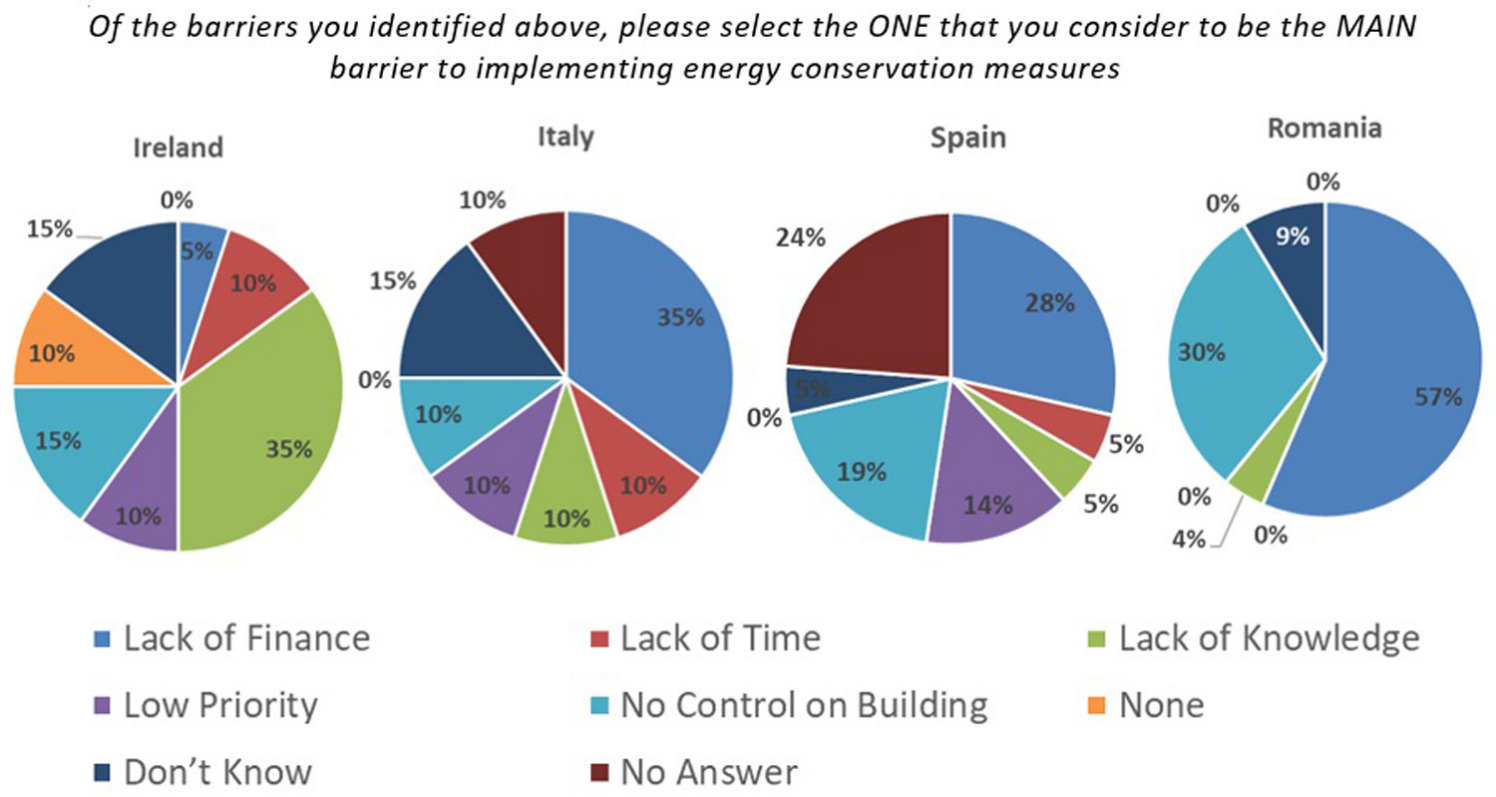
Main challenge for SME to implement Energy Conservation Measure.

One of the European Commission’s Horizon 2020 projects, SPEEDIER, also identified reasons for the low uptake of energy efficiency upgrades at SME level. The SPEEDIER project organised an online survey with SMEs to determine their opinion and attitude towards energy audit and implementation of suggested Energy Efficiency Measures (EEMs). As the result of an online survey, the project team identified a list of barriers/challenges being faced by SMEs that are preventing them to implement energy-efficiency upgrades. Figure 1 summarises the view of SMEs from pilot countries of the SPEEDIER project (Ireland, Italy, Spain and Romania) on the main barriers to implementing EEMs as identified in one of the report ( D2.3) of the project.

From the above figure, it is clear that a considerable percentage of SMEs agreed that lack of finance is one of the major barriers for them to implement suggested EEMs.

When possible, building owners would prefer self-financing to avoid the financing costs. Furthermore, many small energy efficiency projects (<USD 500,000) are difficult to finance because of lack of interest of ESCOs and the high underwriting costs for lenders with respect to the capital investment ( Kats *et al.*, 2011).

Property owners and occupiers take into consideration investments in energy efficiency measures only if they can enjoy their benefits during their occupancy period, therefore the energy efficiency financing mechanism must enable to transfer the repayment obligation to a subsequent owner or occupier ( Van Nostrand, 2011).

Energy efficiency projects reduce the amount of consumed energy and, therefore, the associated energy supply costs. The energy cost savings are accumulated over time and can be used as funding source for subsequent projects. This is the revolving funds approach which is widely used to promote green technologies by the governments. In most of the cases these programmes are started using funds which are borrowed and require that the achieved energy savings cover the loan repayment ( Faghihi *et al.*, 2015).

In the residential sector, the lack of funds for the upfront cost of an energy efficiency retrofit project is one of the key barriers for owners and occupiers in Ireland ( SEAI, 2019). The SPEEDIER project has highlighted that this barrier also exists in the industrial sector and among various types of SMEs.

To help SMEs to overcome these barriers and implement suggested EEMs, the SPEEDIER project recommends an innovative self-financing ‘Ring Fencing Mechanism’. The ring-fencing mechanism eliminates the requirement of initial capital investment for the implementation of EEMs, considering initially no-cost EEMs and using only energy cost savings which have been previously accumulated to fund implementation of new EEMs. Other contractual approaches to implement energy efficiency measures such as green or energy efficiency leases, energy efficiency mortgages, on-bill financing, require monetary expenses since the beginning of the project ( Bird & Hernández, 2012).

## Ring Fencing Mechanism

The Ring Fencing Mechanism is the key innovative feature of the SPEEDIER project, which suggest that businesses, instead of implementing all the recommended EEMs at one go, adopt the staged approach to implement recommended EEMs.

**Figure 2.**
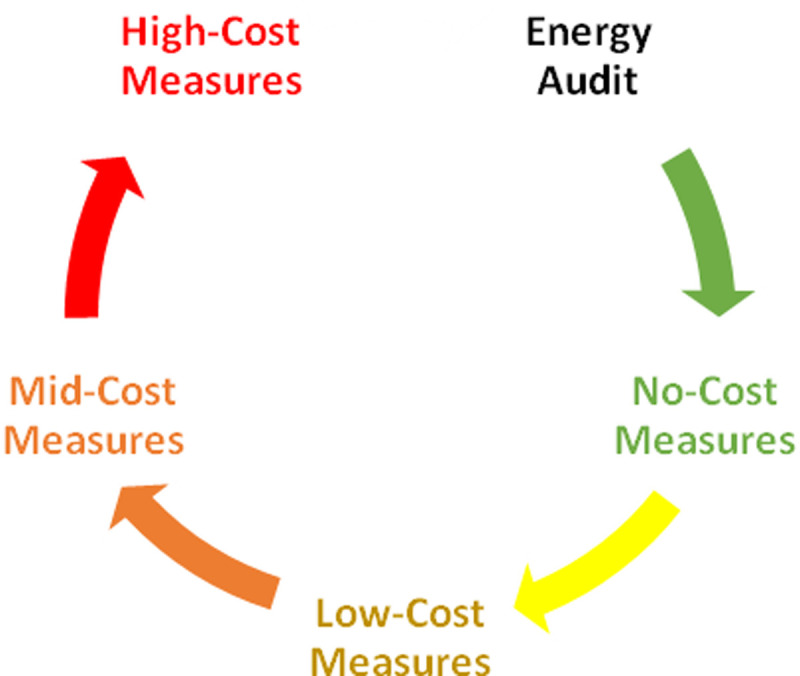
Ring Fencing Mechanism.

In ( Droutsa *et al.*, 2014) it was proposed to prioritize the implementation of EEMs that have low first-cost investment and short payback period. The assumption was the EEMs that give high primary heating energy savings and/or short payback period would be the most attractive for the building owners. Low cost EEMs with short payback period are boiler maintenance, room thermostatic control and solar collectors for 60% domestic hot water. The suggested staged approach proposed in the SPEEDIER project consists in implementing first the EEMs that need zero investment (no-cost EEMs), like reducing thermostat temperature or switching to lower tariff electricity supplier, *etc*. The cost saving realized by implementing no-cost EEMs will be used to implement EEMs that need small investment (low-cost EEMs), then big investment (medium-cost EEMs) and finally bigger investment (high-cost EEMs). To summarize, this funding mechanism recommends starting from implementation of no-cost EEMs first and then ring fence the saving achieved from each stage of ECM implementation for higher investment grade EEMs implementation. In this iterative cycle, cost savings are determined against agreed baseline for each stage of implementation. The achieved savings are reinvested for implementation of additional measures. Figure 2 portrays a simple pictorial representation of ring-fencing mechanism for ECM implementation.

In this way, a business does not need initial investment to start the energy efficiency upgrades. The cost of energy audit and energy expert will also be paid from the saving achieved at each stage of ECM implementation. It is worth to note that the suggested ranking of energy efficiency measures is primarily based on the implementation cost of the measures (because the mechanism aims at eliminating the financial barrier of investment costs in energy efficiency measures), even though other ranking criteria could also be considered within the considered cost groups (sub-ranking criteria), such as the payback period, the CO _2_ emission reduction, the reduction of primary energy use, the investment in renewable energy sources (RES) ( Nikolic *et al.*, 2021). Moreover, this procedure allows the businesses to build their own ‘energy efficiency’ fund within their organization from the savings achieved through staged EEMs implementation without any capital expenditure. This revolving fund encourages businesses to invest and maintain the energy management and efficiency practice of the organization. This continuous ECM implementation process also helps to build energy culture of the organization and creates energy efficiency awareness for their employees.

## Methodology

**Figure 3.**
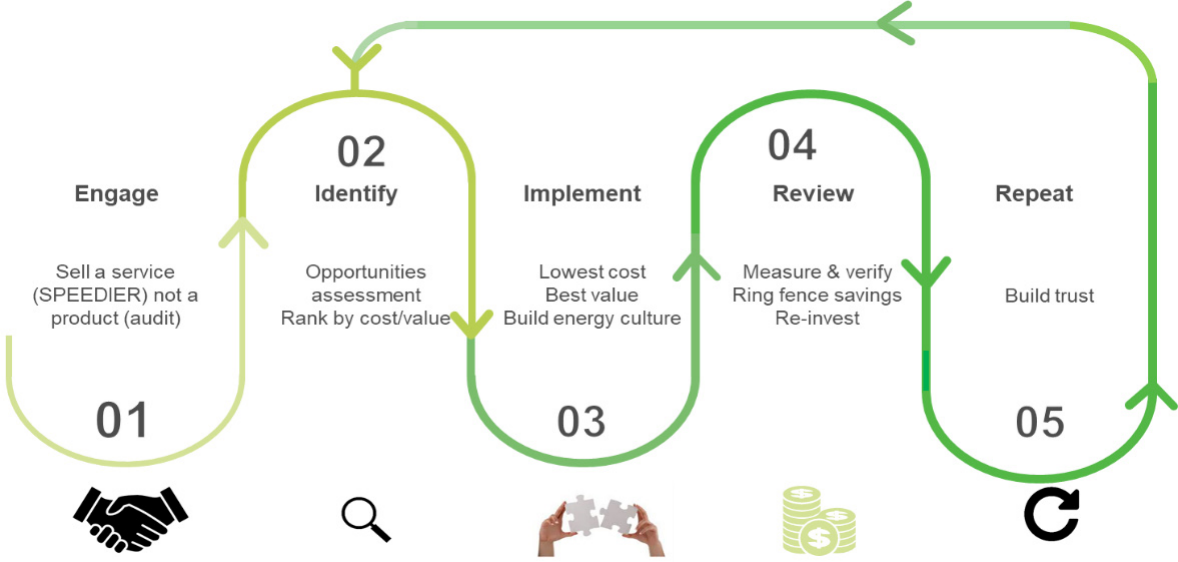
Stages of SPEEDIER Service Implementation.

The ring-fencing mechanism is being implemented within the business through introducing SPEEDIER Service, which has defined several stages of SPEEDIER Service implementation: Engage, Identify, Implement, Review and Repeat. Figure 3 shows a representation of stages of the SPEEDIER service to implement the ring fencing mechanism within any business as explained in one of the report ( D5.2) of the SPEEDIER project.

It is remarked that the energy efficiency service shown in Figure 3 involves a close interaction between the SPEEDIER expert and the business owner during the auditing process. The ring-fencing process for the planning of the energy efficiency measures in Figure 2 is established during the auditing process considering multiple operational aspects which cannot be captured by other planning methods that consider only the implementation costs of the energy efficiency measures, the associated energy savings (and where applicable reduction in CO _2_ emissions), and the budget constraints as the factors determining the choices of the decision maker ( Tan *et al.*, 2016). Approaches for selection of energy efficiency measures relying purely on mathematical formulations which have not been tested within the auditing process have not demonstrated that the benefits are achievable by the SMEs. In fact, several barriers other than the budget affect the actual adoption of energy efficiency measures in SMEs, such as hidden costs, lack of time, possible issues with the procurement of some energy efficiency measures, lack of information, lack of trust in energy efficiency providers, fear of production disruption, organizational aspects of SMEs ( Trianni & Cagno, 2012). Production is given the highest priority in SMEs and energy efficiency measures will be considered only if their installation does not interfere with production processes. For this reason, SMEs are complex organizations where the decision-making process about energy efficiency may involve multiple managers; the production manager almost always has more power and influence than the energy or maintenance manager because of higher priority given production with respect to energy efficiency ( Hasanbeigi *et al.*, 2010). These barriers are identified and managed throughout the auditing process by the SPEEDIER expert and will determine the route taken by the ring-fencing mechanism and the final benefit achieved.

## Case study-1

The first case study will be represented for a business based in Ireland and will be called business-1, hereafter.

### Description of facility

Business-1 is a medium enterprise that produces medical device and technology located in Ireland and manufactures automated machines for industry. There are 220 full-time staff, with 60–70 staff working in offices and 90–100 working in manufacturing sections. The site, which comprises of 33,174 m² total area (including car parks and buildings) and building 1 has 3,779 m² of floor area, building 2 consists of 1,350 m² of floor area and building 3 (purchased in 2021) consists of 2,790 m² of floor area. The total floor area on site is 5,129 m² (2020) and 7,919 m² (2021).

### Baselining the annual energy consumption

The purpose of energy billing analysis is to help understand the site’s yearly usage and how seasonal changes can affect energy usage. There are two main headings: electrical usage and fossil fuel usage. Electrical usage is taken from the on-site Electricity Supply Board (ESB) main incomer and appears on each monthly or bi-monthly billing.

***Electrical usage*.** The final electrical consumption (TEFC) for 2020 is calculated to be 528,410 kWh (EUR 59,443), which is equivalent to 171 tCO₂ emission as shown in Figure 5.

***Thermal usage*.** Natural gas is used as the fossil fuel on site. The monthly billing for period January 2020 to December 2020 was analysed, and the estimated monthly usage was obtained. Natural gas usage accounts for 100% of total thermal final consumption (TTFC) on site and was calculated to be 713,989 kWh FY2019/20, (EUR 42,839) which is equivalent to 146 tCO _2_ emission as shown in Figure 5.

***Total energy breakdown*.** Combining fossil fuel and electrical consumption a breakdown of cost, carbon emissions and total primary energy required. Total Primary Energy Requirement (TPER) is a measure of energy consumption that also accounts for the energy that is consumed and/or lost beyond the boundary of the considered organisation, notably in generating and distributing the electricity that is used by the organization ( Teagasc National Dairy Conference, 2010).

**Figure 4.**
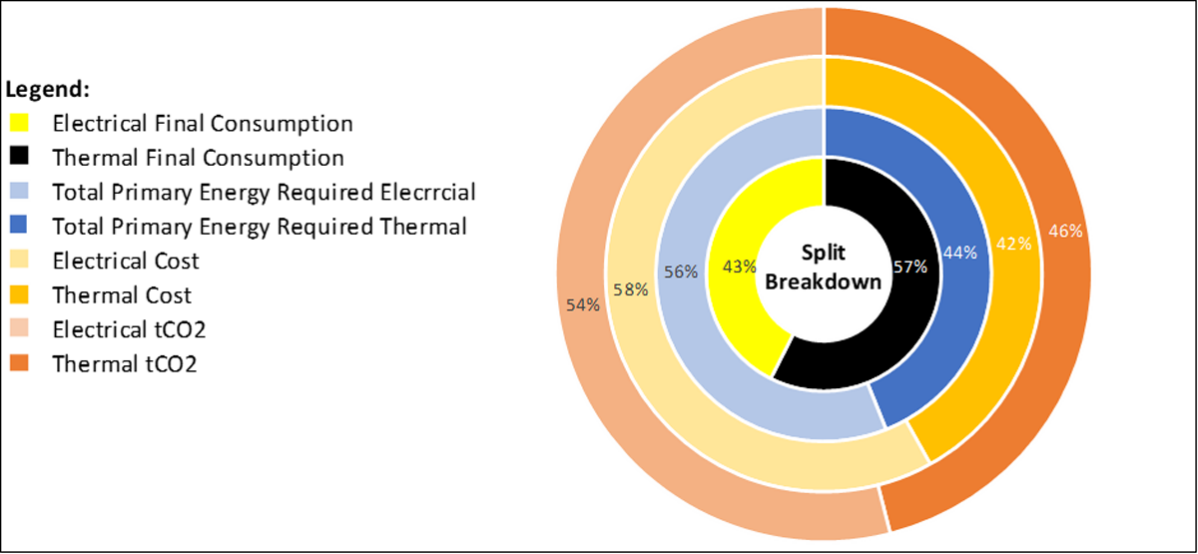
Electrical and Thermal consumption breakdown.

Business-1 has two electricity meters installed. Electricity is responsible for 43% of total site consumption (TSC), with 57% associated consumption to fossil fuels (thermal). Total primary energy demand is split 56:44 with electricity predominantly. This is due to the conversion factor (TPER to Total Final Consumption (TFC)) is 1.89 for electricity, due to transmission losses on the grid, and most fossil fuels having a TPER to TFC conversion factor of 1.1. Cost is broken down 58% electrical, 42% fossil fuel (thermal) and carbon emissions are split 54:46 electricity to fossil fuels (thermal) Figure 4.

**Figure 5.**
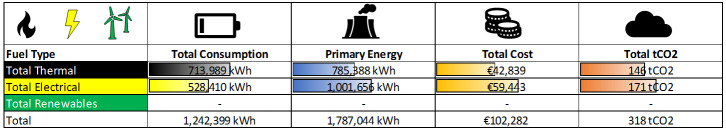
Total energy breakdown FY 2019/20.

Electrical consumption accounts for the largest portion of energy consumption and so can yield the highest savings.

***Energy consuming equipment*.** Building 1 is heated by two gas fired 80 kW boilers (one on standby). Building 1 has a mixture of office sections and manufacturing sections. For the office sections heat is supplied *via* radiators in older section and radiant panels in the newer section. There is a thermostat for the old section of offices and for the new section of offices there are five different thermostats.

All buildings are on a time schedule of 07:00–17:00 h Monday to Friday and 07:00–12:00 h on Saturdays.

Within the office areas of building 1 there are two air handling units (AHU) that ventilate air throughout these spaces. Within the manufacturing areas of building 1, 2 and 3 there are mechanical fan units that circulate air. There are also mechanical fans throughout the toilet facilities. All are operating on a time schedule pre-determined by the BMS system on site.

There are four split air conditioning units that service building 1, three single outdoor units (ODU) provide cooling for the older part of building 1 and one double ODU providing cooling for the new section of building 1. There are other air conditioning split units and chiller units on site but are only used for manufacturing purposes.

Lighting in building 1 consists of 60% fluorescent fittings consisting of 4×22-W FL tubes in office areas and twin 58-W FL tubes in manufacturing areas. The remaining 40% lighting of building 1 consists of 32-W panel LED in office areas and 53-W mega man LED fittings in manufacturing areas.

Building 2 consists of 100% LED in manufacturing areas (same as in building 1) and fluorescent lighting in office areas (same as building 1). Building 3 is 90% fluorescent in manufacturing areas and 100% fluorescent in office areas. All lighting is controlled locally.

There is a large amount of equipment on site, primarily used in manufacturing processes. These other pieces of equipment will not be part of energy measure consideration as the bespoke nature of the business and equipment rules them out. There is a lot of office equipment used on site, including printers, monitors and PCs. These pieces of equipment will be the main focus for a behavioural change energy measure

### Applying the ring fencing mechanism

The SPEEDIER expert suggested a suitable list of energy saving measures to business-1 and presented a five-year implementation plan for same. Some of the EEMs were not selected and agreed by business-1 for the implementation plan. Below is the detailed list of suggested and agreed EEMs along with reason of non-agreement for some of the non-selected EEMs.

***No-cost EEMs*.** Five no-cost EEMs were identified, and business-1 agreed to implement three of them. The EEMs which were not selected are change the electricity supplier and increase the maximum import capacity (MIC) by 30 kVA. Since business-1 had changed their supplier just before the energy assessment so changing electricity supplier would have not been possible. Indeed, the literature has described relational switching costs as the costs which are mainly associated with the loss of personal relationships and emotional bonds with employees of the current energy supplier. In fact, when a relationship with an energy supplier ceases to exist, although no cash goes lost, the outcome of such investments such as time and expenses which have already spent while developing the relationship, are lost ( Walsh *et al.*, 2005).

Moreover, due to the dynamic nature of their business operations (with machines coming online and offline) and future expected energy consumption, the financial controller of the business-1 did not agree to change the MIC. However, the agreed EEMs selected to implement were related to building heating system. The list of selected EEMs is shown below in Table 1:

**Table 1.** No cost energy saving measures for business-1.

No-Cost Energy Conservation Opportunities						
ID	Name	Fuel	Energy Saved / Year (kWh)	Carbon Saved / Year (tCO2)	Cost Saved / Year (€)	CapEx (€)
**B-1**	Reduce Building 1 Boiler Set point	Gas	3,100	1.00	€186	€0
**TS-1**	Reduce Heating time by 1 hour	Gas	71,399	14.62	€4,284	€0
**SP-1**	Reduce Thermostat by 1 Degree	Gas	14,279	2.92	€857	€0
**TOT**	**TOTAL**	**Gas**	**88,778**	**19**	**5,327**	**0**

From Year 1 over €5,327 can be saved and used to implement low to mid cost measures.

**Reduce thermostat set point**

Small changes can start the road to a sustainable future. Reducing the thermostat by 1°C during the heating season within the office and manufacturing areas is estimated to reduce heating costs by 2% as a low estimate, it is stated to reduce energy costs by up to 10% according to BERIreland.ie. As the gas is only being used for heating in the manufacturing floor *via* the two blower units in buildings 1 and 2 and also the office areas *via* radiators the total thermal consumption will be reduced.

**Reduce time schedule of heating**

Another quick and easy savings option is the reduce the number of hours of heating on site. Turning off the heating an hour before close or an hour before that can yield significant savings. A behavioural change campaign to wear jumpers and jacket days accompanies this measure well, as telling staff the reasoning behind the changes, the understanding can be used in their own homes and also encourage people to be more energy efficient.

**Boiler set point optimisation**

If operating temperatures are reduced, typically to 50/30°C, or the temperature differentials can be widened to offer a lower return temperature (80/50°C) then there are significant efficiency gains to be had. A lower return temperature provides greater opportunities for condensing boilers to actually condense. A condensing boiler needs the return temperature to be as low as possible, and at 30°C a condensing boiler would theoretically obtain 95% efficiency. Turning down the boiler set point to 50°C, and reducing the flow rate of the main header pump will optimise the condensing gas boiler.

***Low–mid-cost EEMs*.** Low–mid-cost is any measure under EUR 1000. One low–mid-cost ECM was identified shown below in Table 2:

**Table 2.** Low–mid-cost energy saving measures for business-1.

Low–Mid-Cost Energy Conservation Opportunities						
ID	Name	Fuel	Energy Saved / Year (kWh)	Carbon Saved / Year (tCO2)	Cost Saved / Year (€)	CapEx (€)
**AHU-1**	Reduce Fan speed in AHU in Building 1	Electricity	1,050	0.34	€118	€300
**TOT**	**TOTAL**	**Electricity**	**1,050**	**0.34**	**€118**	**€300**

Total costs for low-cost measures amounts to EUR 300 with yearly savings of EUR 118, (0.34 tonnes of carbon; payback of 3 years) and can be used with no-cost measures to springboard the implementation of high-cost measures.

**Reduce fan speed of air handling unit**

Air handling units are normally designed and sized for maximum occupancy of the area they are ventilating. Occupancy has a huge effect on the amount air changes per hour required, CIBSE guide A: Environmental Design states that 10 L/s per person is the suggested and tried and tested requirement for air handling units. Occupancy-driven air handling units are more energy efficient, less likely to breakdown and provide better control for human comfort levels.

***High-cost EEMs*.** Business-1 was suggested five high-cost EEMs. After a series of conversation and exploring various procurement options they agreed to implement two of them. EEMs which were not agreed are installation of renewable energy generation (PV panel installation), installation of electric vehicle charger and electrification of heating system (heat pump installation). Regarding all of these EEMs, business-1 found none of the procurement options suitable for them. So, business-1 agreed to consider implementation of these EEMs in future and will keep exploring and find a suitable procurement options. High cost is any measure over EUR 1000. The list of selected and agreed to implement EEMs are presented in Table 3.

**Table 3.** High-cost energy saving measures for business-1.

High-Cost Energy Conservation Opportunities						
ID	Name	Fuel	Energy Saved / Year (kWh)	Carbon Saved / Year (tCO2)	Cost Saved / Year (€)	CapEx (€)
**L-1**	Fluorescent to LED Lighting	Electricity	53,009	17.17	€5,958	€21,600
**M&T-1**	Monitor and Targeting System	Electricity, Fossil Fuel	35,699	7.30	€2,142	€7,000
**TOT**	**TOTAL**	**Electricity, Fossil Fuel**	**88,708**	**24**	**8,100**	**28,600**

The discounted payback period of this measure was calculated to be 4 years.

High-cost measures are more focused on carbon abatement rather than cost savings. SEAI grants ( SEAI, 2022) are available for the measures listed which will reduce the payback period.

**Monitoring and targeting**

Energy consumption data must be collected and analysed to understand the potential for energy performance improvement. Targets for energy consumption can then be set and actual energy consumption can be measured against the targets. This, in essence, is monitoring and targeting (M&T). Monitoring and targeting have been found to have multiple benefits for industry, such as:

- Energy cost savings, typically 2–5% due to the ability to identify and rectify excessive consumption as it occurs;
- Improved data for justifying capital investment;
- Improve operations and maintenance (O&M)
- Improved budgeting due to better prediction of future energy consumption;
- Waste avoidance, not just energy, also water and materials;
- Ability to benchmark against competitors and sister companies;
- Measurement and verification of savings from capital investment activities; and
- Compliance with ISO 50001 requirements.

A facility’s energy performance can be better understood by comparing it against similar facilities of the same typology, performing a detailed analysis to identify high intensity of energy consumption, high baseload consumption, energy consumption when the facility is unoccupied on night-time on weekdays, energy consumption when the facility is unoccupied on night-time on weekends, and energy consumption when the facility is unoccupied on weekends. Such a detailed approach supports a better decision-making for energy efficiency investments than the traditional analyses based on aggregate monthly or annual data ( Ferreira & Fleming, 2014).

**Lighting upgrade**

Most areas at business-1 have LED lighting already installed in buildings 1 and 2, but there are some lighting fixtures that are still fluorescent. The acquisition of building 3 has brought an opportunity to carry out a mass change over measure. With building 3 having a good quantity of FL light fixtures. From site visits there was 60% of building 1 with FL fixtures, and 90% of building 3 having FL fixtures.

There is assumed to be 270 fixtures ready to change, with a nominal fixture being the twin 58-W T5. The replacement fixture 53-W LED has a lot of savings attached. An assumed cost per fixture and installation cost brings the project to EUR 20,000–25,000. The yearly savings were calculated at EUR 5,958.21 per year or alternatively 17 tonnes of carbon per year. The discounted payback period of this measure is calculated to be 4.5 years.

If all measures were introduced the site would perform:

- 14% more energy efficient year on year,
- carbon saved will amount to 13% of site carbon emissions,
- and a 13% reduction in energy costs.

**Implementation plan**

**Figure 6.**
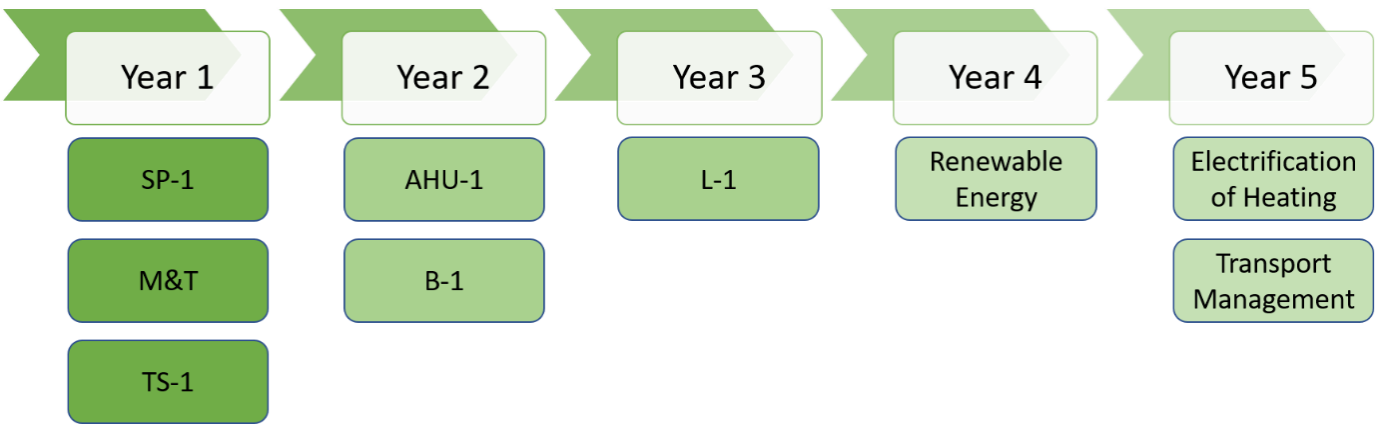
Suggested ECM implementation plan for business-1.

The yearly implementation plan for the next 5 years suggested by the SPEEDIER expert for business 1 is depicted in below Figure 6:

SPEEDIER expert suggested to implement no-cost measures (reduce thermostat set point by 1°C and reduce heating time by 1 hour) in year 1, whereas business-1 agreed to invest their own capital and implement monitoring and targeting system in year 1 as M&T implementation will yield more savings in coming years. Set-point temperature of thermostat has a significant impact on heating (and cooling) energy consumption ( Moon & Han, 2011). In year 2, business-1 agreed to implement one no-cost measure (reduce boiler set point by 1°C) and one low-cost measure (reduce fan speed of AHU in Building 1).

Business-1 wanted to reduce the boiler set point temperature by 1°C after getting a full understanding of energy usage and pattern through installed monitoring and targeting system in year-1. In year 3, business-1 agreed to implement lighting measure and change the fluorescent bulbs with LEDs of same lux level.

**Figure 7.**
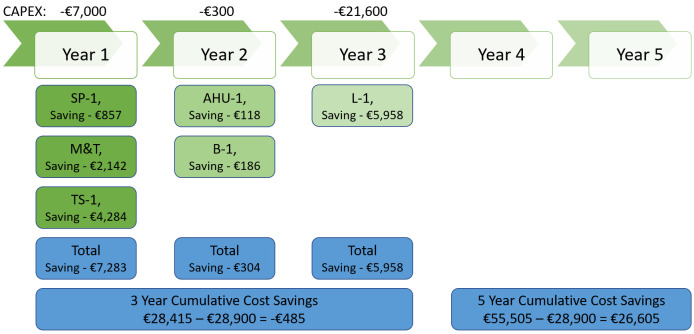
5 year implementation plan - cost saving for business-1.

Business-1 also agreed to installation of renewable energy generation (PV panel installation), installation of electric vehicle charger and electrification of heating system (heat pump installation) in years 4 and 5. But they didn’t find any suitable quotation and procurement options at this point of time to provide commitment for ECM implementation, indeed they agreed keep exploring a suitable quotation and implementation of these EEMs in future. Business-1 faced the barrier of hidden costs (lack of time and/or management costs) associated with the procurement of PV-panel and heat pump measures ( Schleich, 2007). So, Figure 7 represents a 5-year plan for ECM implementation along with investment and cost savings for business-1 without considering high-cost measures like renewable energy installation, transport management and electrification of heating. From Figure 7, it is clear that business-1 will need to invest EUR 28,900 for initial 3 years and by the end of year 5 they will be saving EUR 26,605.

**Figure 8.**
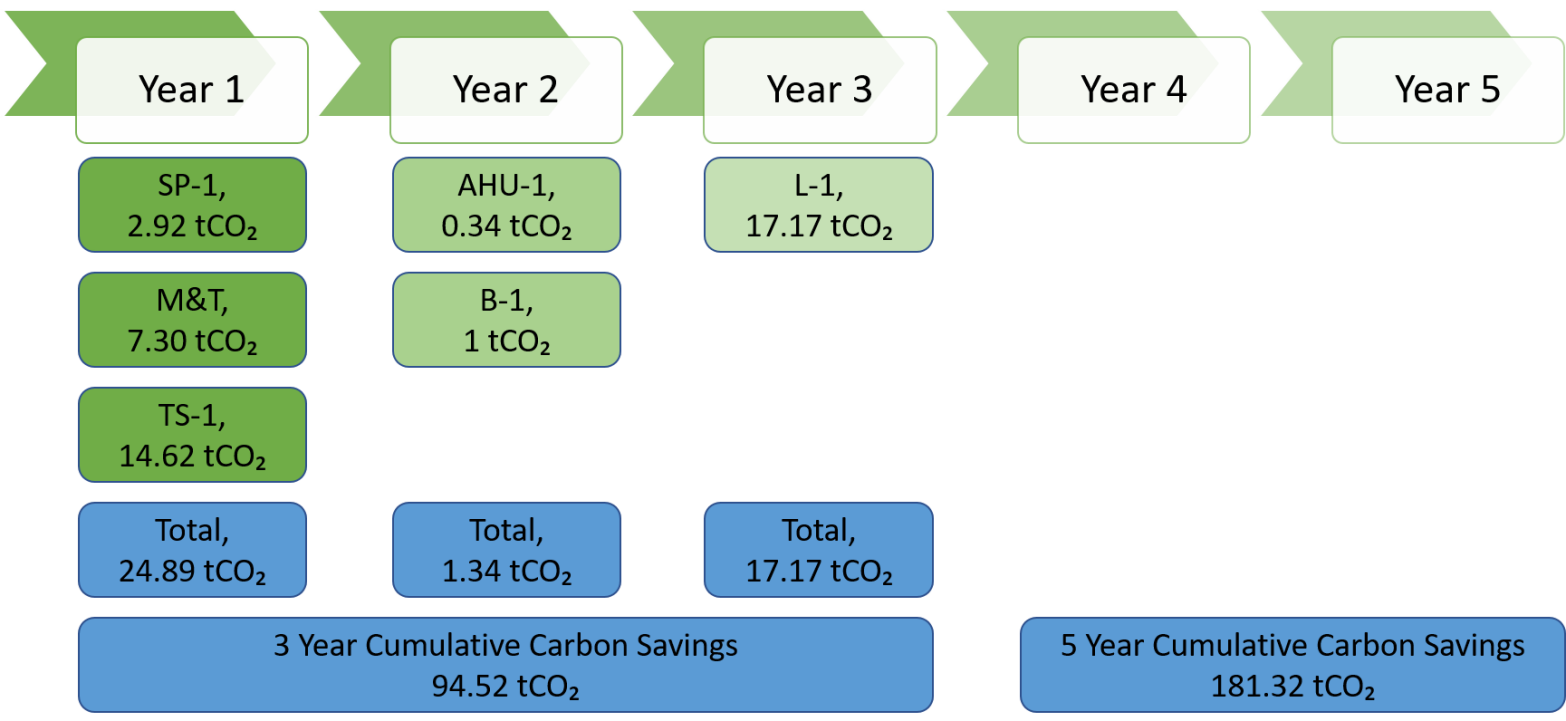
5 year implementation plan - carbon saving for business-1.

Below Figure 8 depicts carbon savings to be achieved in next 5 years, from the picture it is clear that by the end of year 5, business-1 will curb 181.32 tCO₂ greenhouse gas emission.

## Case study 2

The second case study is a business based in Ireland and will be called business-2, here after. 

### Description of facility

Business-2 is a goat’s cheese manufacturing small enterprise based in Ireland, which has been producing cheese since 1999 from their manufacturing plant. There are five full-time staff; and during the summertime the staff can increase to 15. Manufacturing is the main type of end use on site. In 2019, business-2 produced 35 tonnes of product, from an average herd of 300 goats.

### Baselining the annual energy consumption

The purpose of energy billing analysis is to help understand the site’s yearly usage and how seasonal changes can affect energy usage. An electricity meter, 20 solar PV panels and fossil fuels are used to satisfy the energy needs of business-2. 

***Electrical usage*.** Business-2 has an annual total electrical final consumption (TEFC) of 72,505 kWh accounting for 64% of total site final consumption (TSFC). The solar panels produce 3.6% of imported electricity with scope for further increasing the PV array. Electricity for 2019 was calculated to be 69,967 kWh (EUR 12,470), which is equivalent to 23 tCO _2_ emission as shown in Figure 10.

***Thermal usage*.** Fossil fuels are provided by kerosene and LPG boilers with an annual total thermal final consumption (TTFC) of 40,822 kWh (36% of TSFC). It is noted that on-site there is a 1000-L LPG bulk tank unconnected to the LPG boiler, hence why there are bottled LPG deliveries throughout the year. Kerosene usage on site accounts for 96% of total fossil fuel usage on site for year 2019, with 4% accounting for LPG.

***Total energy breakdown*.** Combining fossil fuel and electrical consumption (with supplementary PV array) a breakdown of cost, carbon emissions and total primary energy required as shown in Figure 10.

**Figure 9.**
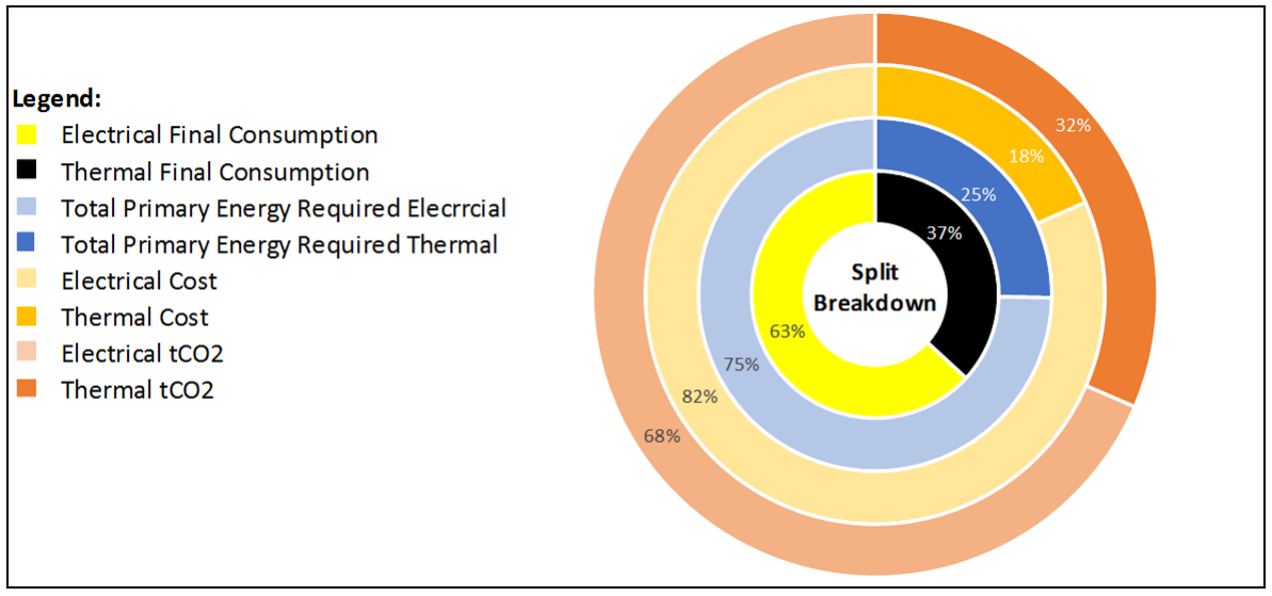
Electrical and thermal consumption breakdown for business-2.

Electricity is responsible for 63% of total site consumption (TSC), with 37% associated consumption to fossil fuels (thermal). Total primary energy requires is split 75:25 for predominantly electricity. This is due to the conversion factor (TPER to TFC) is 1.89, due to transmission losses on the grid, and most fossil fuels having a TPER to TFC conversion factor of 1.1. Cost is broken down 82% electrical, 18% fossil fuel (thermal) and carbon emissions are split 68:32 electricity to fossil fuels (thermal) as shown in Figure 9.

**Figure 10.**
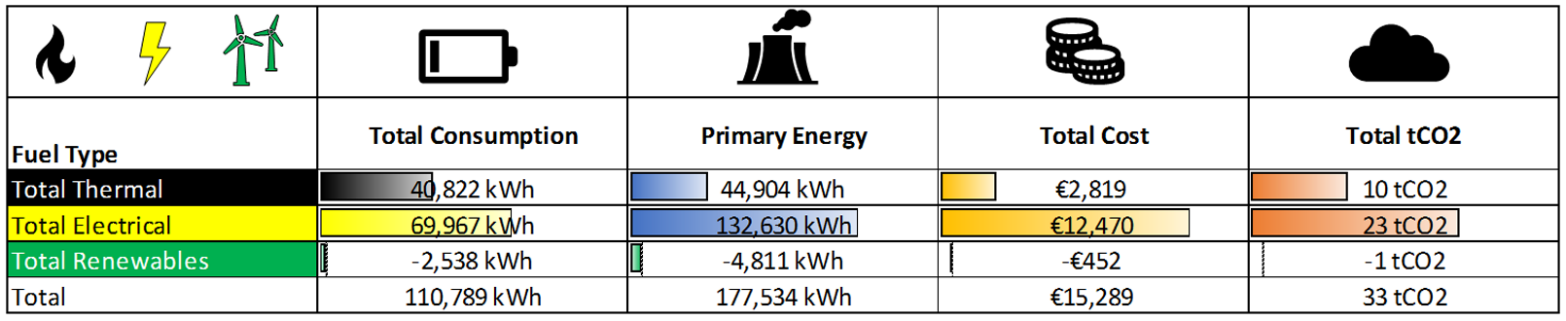
Total energy breakdown FY19 for business-2.

Electrical consumption accounts for the largest portion of energy consumption and so can yield the highest savings.

***Energy consuming equipment*.** The main building is heated by a single fire bird kerosene boiler, estimated to be 26 kW. Some of the spaces are heated *via* underfloor heating at a set point of approximately 20°C. Hot water is heated *via* an ACV cylinder with approximately 500-L capacity at a set point of 60°C.

Space heating is provided *via* under floor heating (60 m²). This system is temperature controlled and time controlled. It was noted that the thermostats for space heating were faulty or in need of repair. There was a substantial insulation project undertaken within the last 3 years that focused primarily on cavity insulation and attic insulation.

There are seven domestic hot water outlets that see sporadic use throughout a working week. The time schedule for hot water is set to Monday to Friday (6:30–08:30 h, 12:00–15:00 h) and Saturday and Sunday (07:30–10:00 h). One of these outlets (milking machine) uses an electric element for domestic hot water and is used typically twice a day for 10 minutes, Monday to Friday.

Process hot water is heated by an 18 kW electric immersion for use in the pasteurising process typically used for 3 times a week for 2 hours. Changes in pasteurization are process related energy efficiency measures, which can be applicable to specific dairy processes.

There is no air conditioning for comfort cooling on site. There are direct expansion (DX) units on site, but these units are used for the ice bank, fridges, and freezers, and four cold rooms. The ice bank is cooled by a DX unit; cold water then chills milk from the milking machine *via* a plate heat exchanger.

Lighting is a mix of surface mounted fluorescent tube fittings and surface mounted LED fittings. The yearly lighting energy is assumed to be 700 kWh. The lights are locally controlled *via* ON/OFF light switches.

There is a large amount of equipment on site, primarily used for cheese manufacturing. The list of equipment is shown below in Table 4:

**Table 4.** List of energy consuming equipment for business-2.

Equipment	Location	Description	Rated Power (kW)	Estimated kWh FY19
**Armour 1**	Packing room	Humidity control, cheese process – 1000 W from internet	1	2,100
**Armour 2**	Packing room	Humidity control, cheese process – 1000 W from internet	1	2,100
**Cold room fridge 1**	Packing room	Kelvion Searle TEC3.5-5 cooler (Single Phase) as seen on site	1.11	2,016
**Cold room fridge 2**	Packing room	Kelvion Searle TEC3.5-5 cooler (Single Phase) as seen on site	1.11	2,016
**Cold room fridge 3**	Packing room	Kelvion Searle TEC3.5-5 cooler (Single Phase) as seen on site	1.11	2,016
**Free-standing fridge 1**	Packing room	Typical Fridge Power	0.8	700
**Vacuum packing machine**	Packing room	From Nisbets Catalogue	0.9	700
**Mixing machine**	Warm room 1	From Nameplate	3	3,150
**Bulk tank 1**	Dairy	N/A	-	5,000
**Bulk tank 2**	Dairy	N/A	-	5,000
**Clothes Washing machine**	Lean-to	Typical Clothes Washing Machine Power	0.255	1,050
**Clothes dryer**	Lean-to	Typical Clothes Dryer Machine Power	2.79	435
**Ice Bank**	Dairy	When used with pasteurised cheese, used to cool product from 60°C to 4°C. When used with raw cheese, 40 to 4 to 24. 150 L to 600 L/day	N/A	11,368
**Pasteurizer**	Dairy	Unison. Estimate 100 goats at 2.5 L/day processed in 2 hours, = 125 L/h. heated from 20 to 70°C	N/A	5,075
**Dishwasher**	Washroom	Typical Commercial Dishwasher Power	2.4	700
**Fan on extract hood above** **D/W**	Washroom	Typical Extractor Fan Power	0.5	175
**Dispatch fridge, exterior condenser**	Dispatch room	Typical Fridge Power	0.8	2,016
**Computer**	Office	Typical PC Power	0.2	175
**Printer-scanner**	Office	Typical Printer Scanner Power	0.5	35
**Canteen Chest freezer**	Canteen	Leibherr, 1.5 A	0.4	483
**Canteen Upright freezer**	Canteen	Typical Fridge Power	0.8	483
**Canteen fridge**	Canteen	Typical Fridge Power	0.8	483
**Toaster**	Canteen	Typical Toaster Power	1	35
**Kettle**	Canteen	Typical Kettle Power	2	1,575
**Lighting**	All areas	Assumed Load (20 FL T8s @ 72 W, and 10 LED @36 W)	1.8	700
**Milking machine**	Dairy	3-phase, 2 vacuum pumps fixed speed, 2×3 kW motor, 1-1.5 hours, twice per day	6	2,415
**Compressor**	Dairy	for auto-clusters, ration dispenser, pasteuriser, depositor. 1 hour per day, 2.2 kW	2.2	770
**Milk replacer feeding m/c**	Dairy	single phase electric heating element, 3 kW. Used for 1 month.	3	4,200
**Milking machine washer**	Dairy	2×3 kW immersions. 150 L tank. 2×90L per day at 85°C	6	5,481
**TOTAL**	**All areas**		**41.475**	**62,452**

### Applying the ring fencing mechanism

***No-cost EEMs*.** Four no-cost EEMs were identified and out of four suggested EEMs changing energy supplier was not agreed because they switched the energy supplier just before the SPEEDIER expert performed energy assessment at business-2. The list of selected EEMs is show below in Table 5

**Table 5.** List of no-cost EEMs for business-2.

No-Cost Energy Conservation Opportunities						
ID	Name	Fuel	Energy Saved / Year (kWh)	Carbon Saved / Year (tCO2)	Cost Saved / Year (€)	CapEx (€)
**DX-1**	Night-time Cooling	Electricity	2,514	0.82	€1,015	€0
**PHE-1**	Plate Heat Exchanger (PHE) Optimisation	Electricity	6,639	2.15	€1,183	€0
**WIF-1**	Walk-in Fridge: Evaporator Cleaning	Electricity	302	0.10	€54	€0
**TOT**	**TOTAL**	**Electricity**	**9,455**	**3**	**€2,646**	**€0**

From year 1, over EUR 2,500 can be saved and used to implement low cost measures.

**Night-time cooling**

Assuming that the ice box is used only when it is needed. The ice box should be placed on a timer, so that it only cools at night. This will reduce costs by taking advantage of the cheaper night rate and saving energy by taking advantage of the cooler temperatures during the night ( Ireland Climate overview, 2021).

**PHE optimisation**

PHE ratio between milk and water is below 1:3, the cooling potential of the PHE is not being met. To reduce the cooling requirement, ensure that 3 times the amount of water enters the PHE. This can reduce cooling times, and reduce energy used by both pumps ( Teagasc National Dairy Conference, 2010).

**Walk-in fridge: Evaporator cleaning**

Walk in fridges and Freezers need regular cleaning (2–3 a month) making sure that the evaporators are not being blocked. It also ensures that only the product is cooled. Fridges should have enough room for the air to circulate within, while freezers should be filled to about 70% to reduce the amount of air to cool ( Die-Pat, 2022).

***Low–mid-cost EEMs*.** Low–mid-cost is any measure under EUR 1000. There are five low–mid-cost EEMs were identified and agreed to implement as shown below in Table 6:

**Table 6.** List of low–mid-cost EEMs for business-2.

Low–Mid-Cost Energy Conservation Opportunities						
ID	Name	Fuel	Energy Saved / Year (kWh)	Carbon Saved / Year (tCO2)	Cost Saved / Year €)	CapEx (€)
**H-1**	Thermostat replacements	Electricity	655	0.21	€173	€52
**PHE-2**	PHE Insulation	Electricity	1,421	0.46	€253	€500
**LPG-1**	LPG Boiler Upgrade	Fossil Fuel	7,820	3.09	€2,745	€1,000
**WIF-2**	Walk in Fridges Seal	Electricity	1,028	0.33	€2,932	€500
**DHW-1**	Domestic Hot Water Optimisation	Fossil Fuel	1,028	0.33	€2,932	€25
**TOT**	**TOTAL**	**Electricity,** **Fossil Fuel**	**4,132**	**2**	**€ 9,035**	**1,637**

Total costs for low-cost measures amounts to EUR 1,637 with yearly savings of EUR 9,000, (4.42 tonnes of carbon, payback 3 months) and can be used to springboard the implementation of high-cost measures.

**PHE insulation**

Insulation of PHE and piping is essential to reducing heat loss (in heating systems) and heat gain (in cooling systems) inefficiencies throughout the system ( Alfa Laval, Gasketed Plate Heat Exchanger, 2021).

**Walk-in fridge sealing**

Seals on the walk-in fridges and freezers should be inspected for tears and replaced. Alarms (like leap sensors) should also be placed on each to identify when the temperature has gone over the optimal cooling temperature and how often ( Brush, 2012; Die-Pat, 2022; Xu *et al.*, 2009; Xu & Flapper, 2009).

**LPG boiler upgrade**

There is already an LPG boiler and an LPG 1000 L bulk tank on site. LPG has a lower g CO _2_ per kWh than kerosene ( Brush, 2012; SEAI conversion factors, 2019).

**Domestic hot water optimisation**

Conventional wand wash basin aerators use more water than required, specialised aerators can save you up to 75% water compared to using the standard aerator ( SEAI, 2019). When using hot water this can significantly reduce the amount of hot water required for hand washing, washing down areas which in turn will reduce the need for the boiler to heat water.

***High-cost EEMs*.** High-cost is any measure over EUR 1000. Two high-cost EEMs were identified ( Table 7).

**Table 7.** List of high-cost EEMs for business-2.

High-Cost Energy Conservation Opportunities						
ID	Name	Fuel	Energy Saved / Year (kWh)	Carbon Saved / Year (tCO2)	Cost Saved / Year (€)	CapEx (€)
**VSD-1**	VSD Installation	Electricity	3976	1.29	€708	€2,000
**MNT-1**	Monitor and Targeting System	Electricity, Fossil Fuel	5539	1.46	€764	€2,000
**TOT**	**TOTAL**	**Electricity, Fossil Fuel**	**9515**	**3**	**€1,472**	**€4,000**

High-cost measures are more focused on carbon abatement rather than cost savings. SEAI grants are available for the measures listed which will reduce the payback period. However, with the savings from No cost, low cost, and mid cost measures are double that of capital expenditure. In addition to the selected measures shown in Table 7, although more expensive, the combined heat power (CHP) generation is considered by the dairy industry the main measure to achieve energy efficiency goals ( Ramirez *et al.*, 2006).

**VSD Installation**

Variable speed drive control is a common measure used in a variety of food processing industries ( Brush, 2012; Xu *et al.*, 2009; Xu & Flapper, 2009). A speed control can deliver economic benefits for about half of all the electric drives used in the mechanical engineering and food industry ( Javied *et al.*, 2016).

Programs based on this measure along with the leak repair in steam systems and refrigeration systems may result in significant specific energy consumption reductions in the cheese-making industry. Reductions in electricity consumption from 5% to 35% can be achieved using VSD ( Han & Yun, 2015). A reduction of more than 9% in the Netherlands for cheese-making industry from 1998 to 2002 was reported in ( Xu *et al.*, 2009).

Conventional vacuum systems for milking machines incorporate a vacuum pump operating at a fixed speed, a vacuum regulator, and a load. The load consists of the air admitted by the components that make up the milking system including milking units, clusters and other devices that admit air during operation. The load is not constant so therefore the speed vacuum pump should vary depending on the load required.

**Monitoring and targeting system**

**Figure 11.**
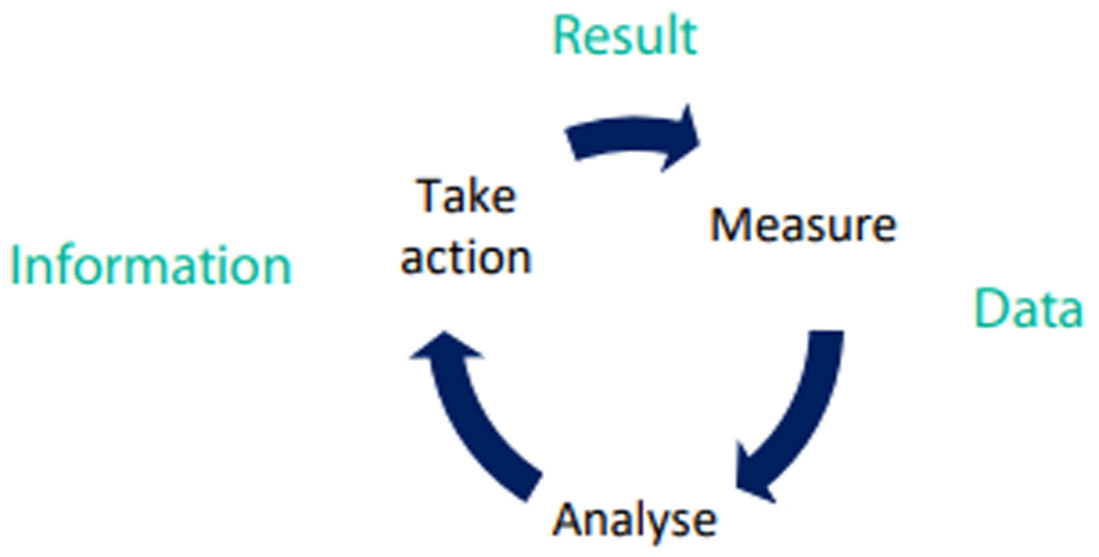
Monitoring and Targeting System.

Energy consumption data must be collected and analysed to understand the potential for energy performance improvement. Targets for energy consumption can then be set and actual energy consumption can be measured against the targets. This, in essence, is monitoring and targeting (M&T). A pictorial representation of M&T system is shown in Figure 11.

Monitoring and targeting has been found to have multiple benefits for industry, such as:

• Energy cost savings, typically 5–15%, due to the ability to identify and rectify excessive

consumption as it occurs;

• Improved data for justifying capital investment;

• Improved budgeting due to better prediction of future energy consumption;

• Waste avoidance, not just energy, also water and materials;

• Ability to benchmark against competitors and sister companies;

• Measurement and verification of savings from capital investment activities; and

• Compliance with ISO 50001 requirements.

### Implementation plan

**Figure 12.**
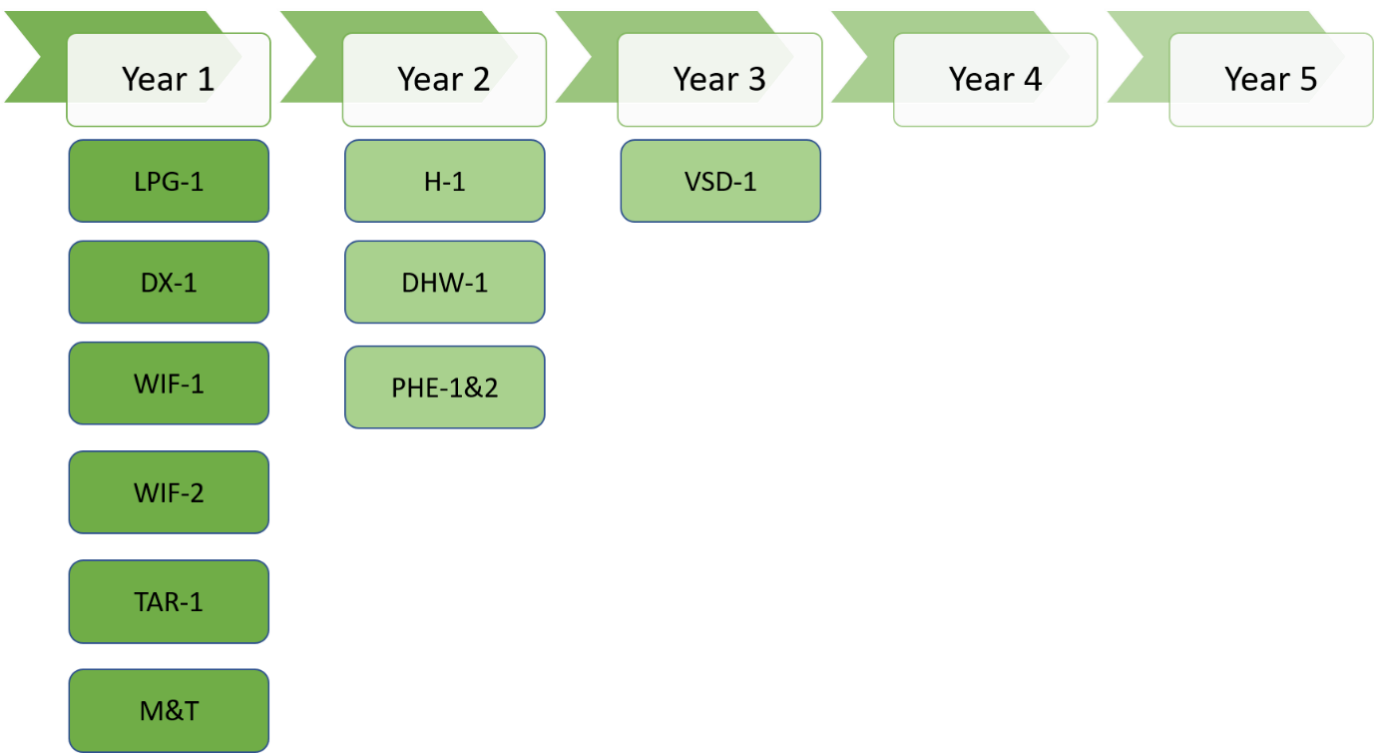
Suggested ECM implementation plan for business-2.

The yearly implementation plan for the next 3 years suggested by the SPEEDIER expert for business-2 is depicted in Figure 12:

The SPEEDIER expert suggested to implement no-cost measures (night-time cooling, PHE optimization, Walk-in fridge evaporator cleaning and change energy supplier) in year 1, low-mid cost measures (thermostat replacement, LPG boiler upgrade, PHE insulation, walk-in fridge sealing replacement, and domestic hot water optimization) in year 2 and high-cost measures (monitoring & targeting system and VSD installation) in year 3.

Business-2 agreed to invest their own money as capital cost for some of the low–mid-cost and high-cost measures in year-1 and suggested to implement measures related to plate heat exchanger (PHE) altogether in year-2 (although suggested PHE optimization is no cost measure). After understanding the benefits and importance of monitoring & targeting system, business-2 also agreed to invest their capital cost and install monitoring and targeting system in year 1. Changing energy supplier was not agreed because they switched the energy supplier just before the SPEEDIER expert performed energy assessment.

So, in year-1, business-2 agreed to implement below EEMs: -

1. Boiler replacement (low-cost)
2. Night time cooling (no-cost)
3. Walk in fridge evaporator cleaning (no-cost)
4. Walk in fridge sealing replacement (low-cost)
5. Monitoring & targeting system (high-cost)

In year 2, business-2 agreed to implement some low cost and some no cost measures. So, in year 2 business-2 will implement below EEMs: -

1. Thermostat replacement
2. Domestic hot water optimization
3. Plate heat exchanger optimization
4. Plate heat exchanger insulation

Business-2 didn’t agree to install VSD because they are planning to replace the pump unit in the coming years, hence investing for VSD installation would be redundant by the time of pump replacement. 

**Figure 13.**
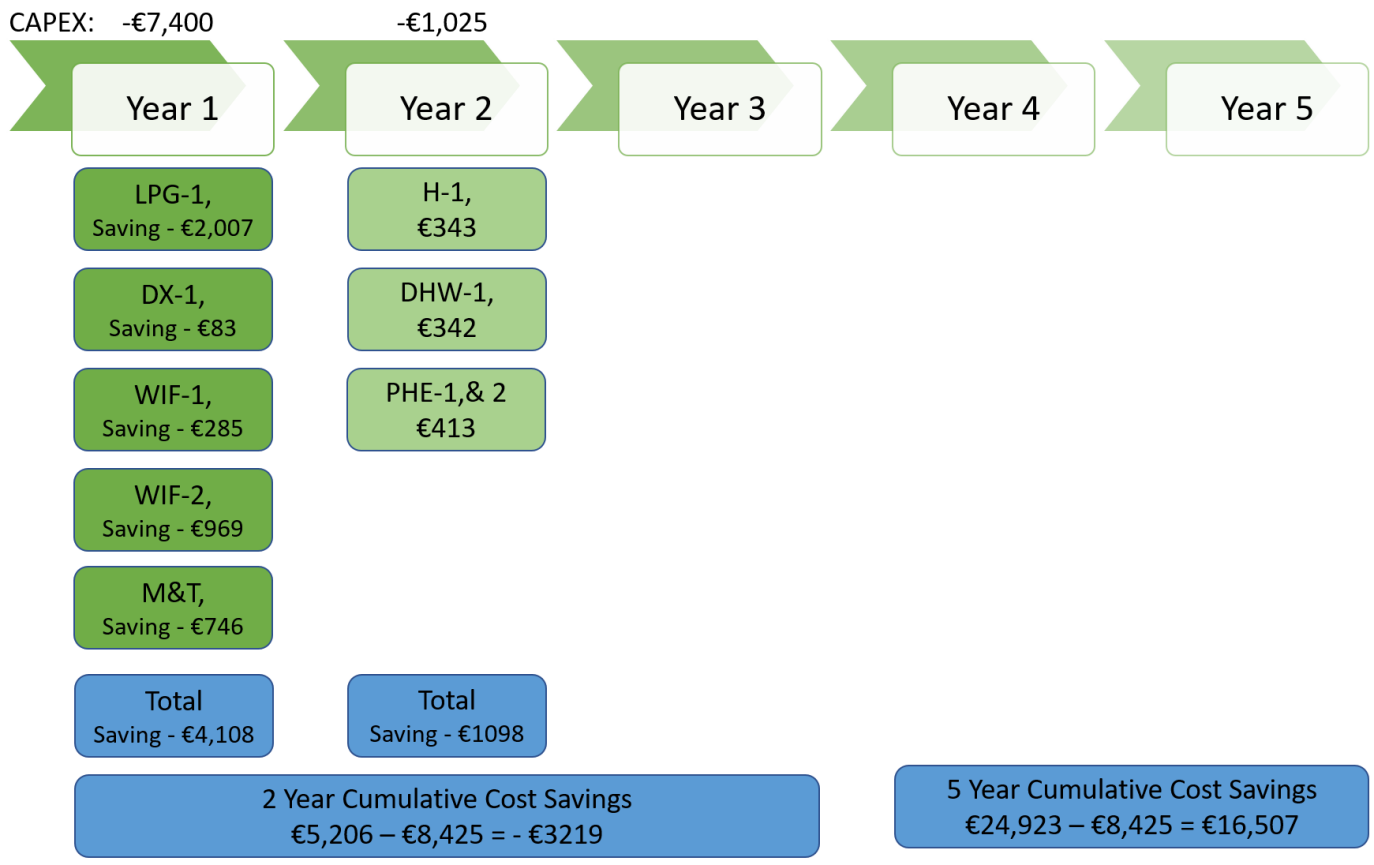
5 year implementation plan - cost saving for business-2.

Figure 13 represents a 5-year plan for ECM implementation along with investment and cost savings for business-2 without considering high-cost measures like VSD installation. From the picture, it is clear that business-2 will need to invest EUR 8425 in 2 years and then, by the end of year-5, business-2 will save EUR 16,507 against their energy cost.

**Figure 14.**
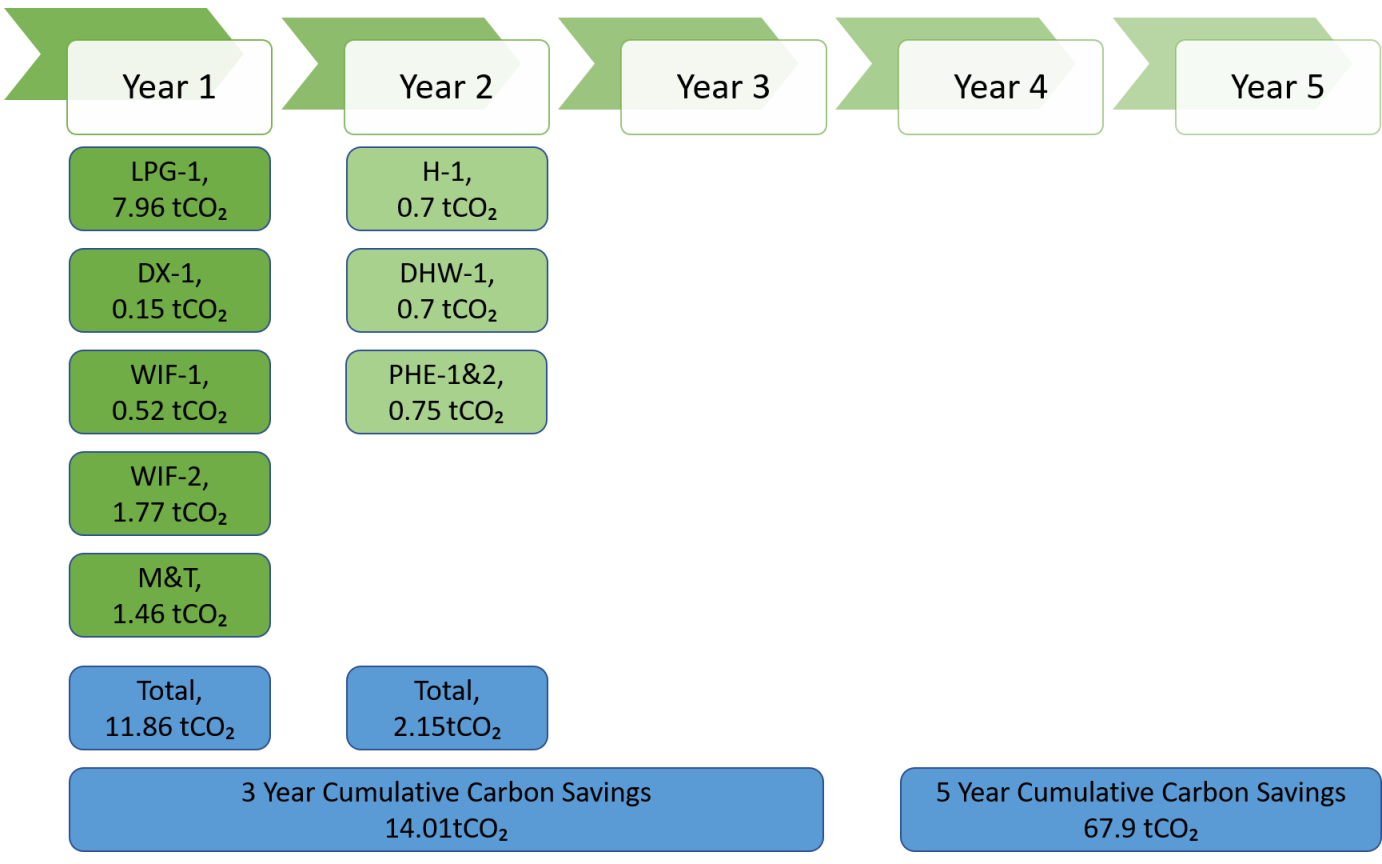
5 year implementation plan - carbon saving for business-2.

Figure 14 depicts carbon savings to be achieved in next 5 years and from the picture it is clear that by end of year-5, business-2 will reduce greenhouse gas emissions by approximately 68 tCO₂.

## Case study-3

**Figure 15.**
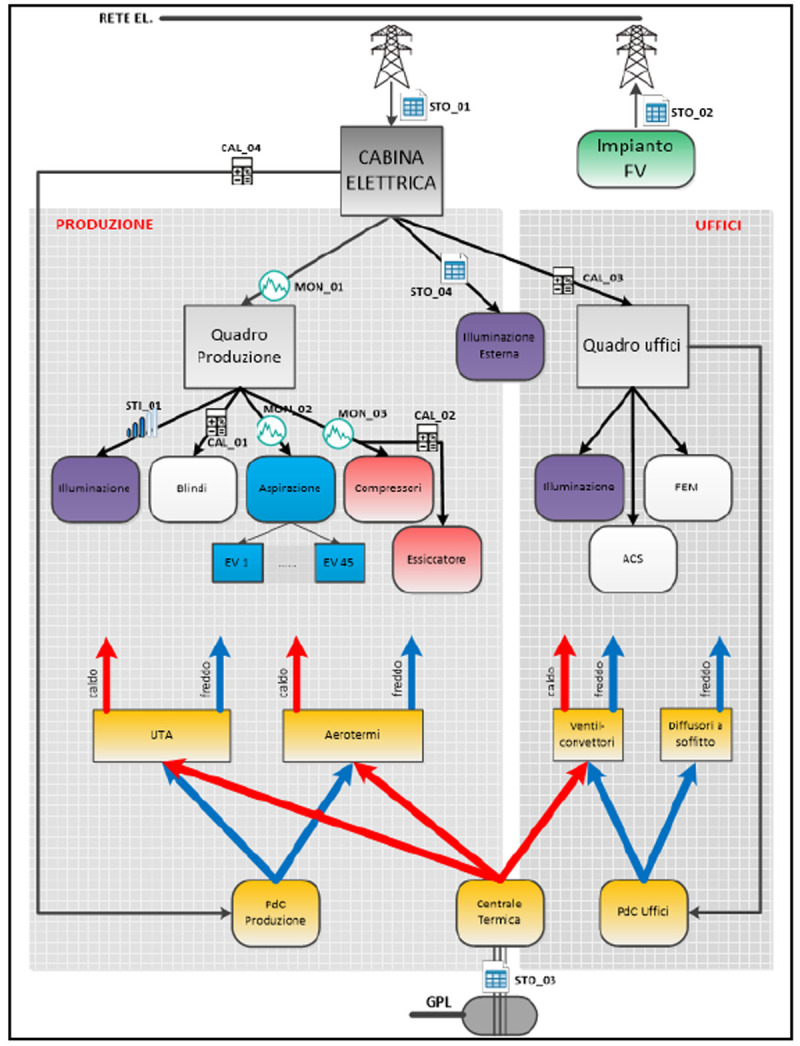
Monitoring system implemented by business 3.

The third case study refers to a small business in central Italy with revenues between EUR 2 million and EUR 5 million. The business owner engaged with the SPEEDIER expert to undergo through an energy audit. They have implemented a temporary monitoring system ( Figure 15) to be used throughout the audit process for the baseline energy consumption data acquisition and at a subsequent stage as the means for energy performance monitoring and energy savings evaluation. The adoption of a monitoring systems enables to identify users’ behaviours with respect to electric load usage and to perform load shifting from peak periods to off-peak periods of the electricity tariff. Most advanced monitoring systems enable to identify loads connected to the electricity network from a single aggregate current waveform, considering the fingerprints of the loads’ current waveforms previously measured and stored in the system and applying an algorithm which can detect correlation, such as the Pearson's correlation or an artificial neural network ( Araújo Kuhn Pereira *et al.*, 2020). In case study 3, the monitoring system was owned by the ESCO and offered with a pay per use formula, which means that the customer agreed to pay a monthly fee to the ESCO for using the monitoring system to obtain the measurements of interest. This solution facilitates investment of SMEs into an energy monitoring system because it overcomes the high costs of purchasing the software and provides a partnership with a company which can effectively support the operative and strategic decision making as well as the planning and controlling processes ( Rackow *et al.*, 2015).

**Figure 16.**
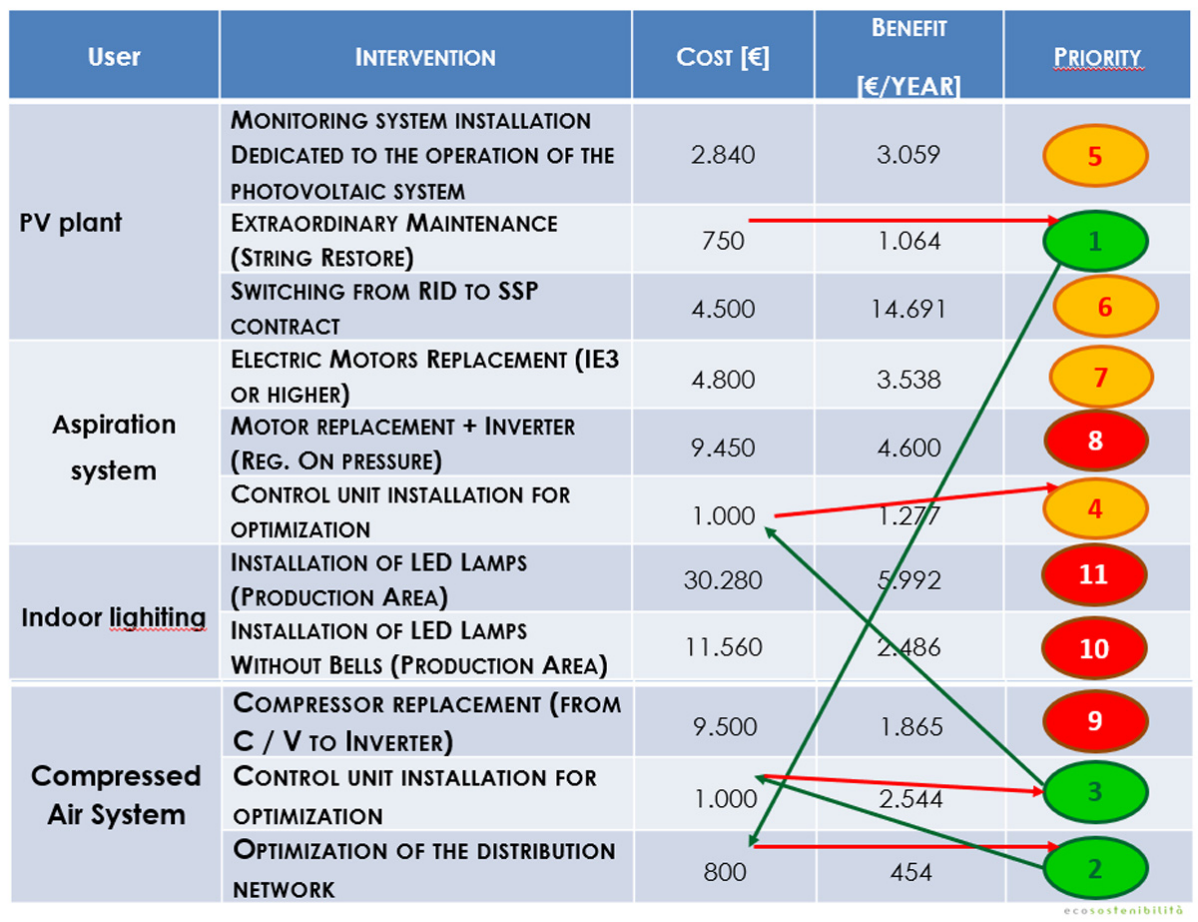
Energy Efficiency measures considered and implemented using the ring-fencing method.

The auditing process followed with the SPEEDIER expert has comprised a five-step process. Initially, the business owner and the SPEEDIER expert have defined the scope of the auditing (step 1). After that an energy assessment including the billing analysis and the creation of a register of opportunities was performed (step 2). The selection of wanted energy efficiency measures (EEMs) was followed by their implementation (step 3). With the EEMs installed, energy consumption was monitored to evaluate the savings with respect to the baseline energy consumption (step 4). After evaluating the benefits, the business owner with the help of the SPEEDIER expert decided whether to repeat the whole process from the first step (step 5). The SPEEDIER auditing and consultancy service was delivered with the agreement that if the business owner decided not to execute the energy efficiency plan, he should be obliged to pay a consultancy fee. On the other hand, if the energy efficiency savings are obtained, they can be used to repay the consultant. This represents a strong incentive for the customer to commit with the energy efficiency project execution. Eleven EEMs were considered ( Figure 16). They may be grouped in four categories. The first group includes improvements to the operation of the existing PV plant. The second group concerns maintenance and upgrades of the aspiration system. The third group is about upgrades of the lighting system using LED lamps. The fourth group includes renovation measures applicable to the compressed air system and air distribution network.

**Figure 17.**
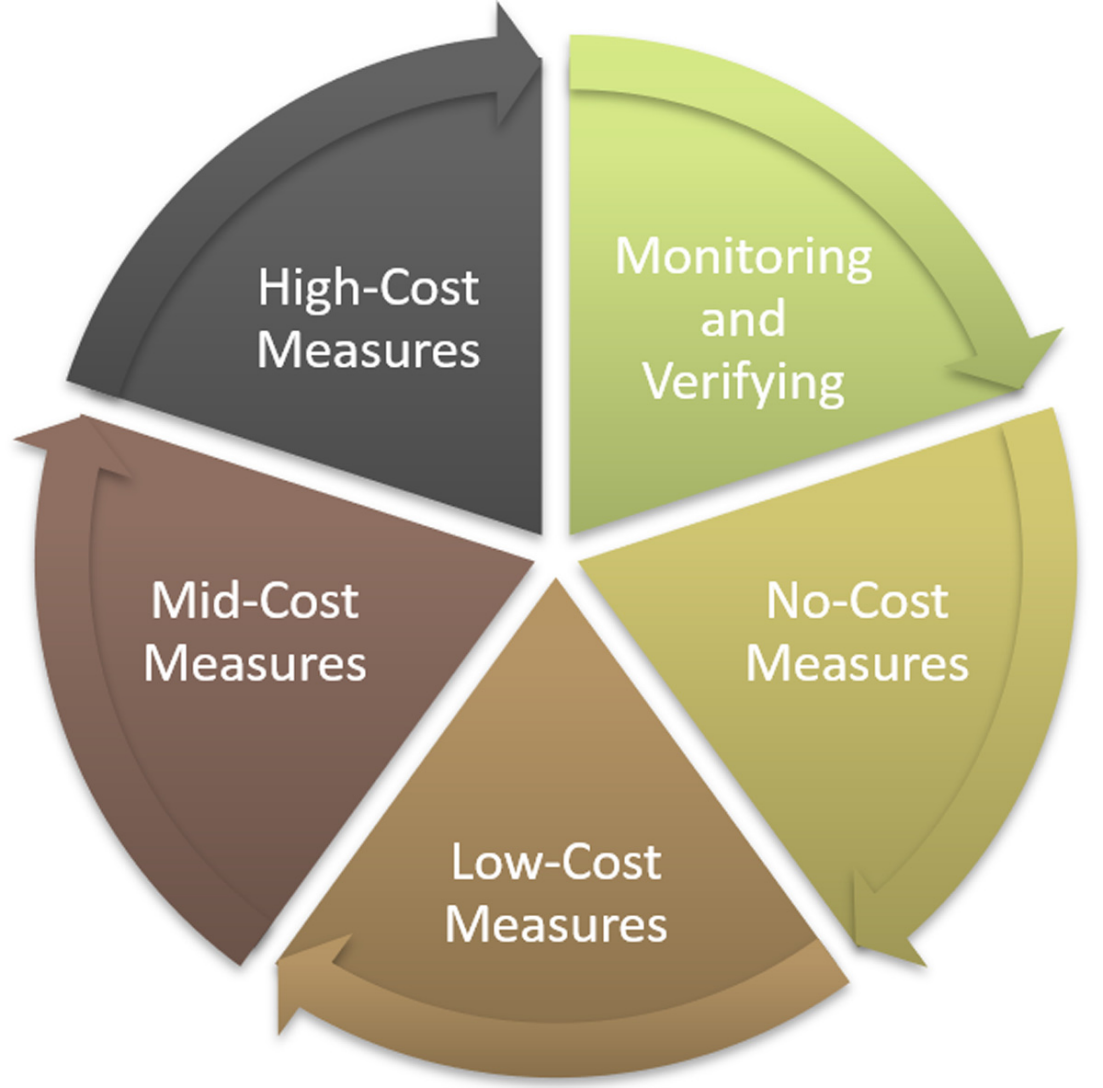
Process followed by business 3 to implement energy efficiency measures using SPEEDIER ring fencing methodology.

To select the most appropriate energy efficiency measures, the SPEEDIER expert followed the ring-fencing approach illustrated in Figure 17. The SPEEDIER ring-fencing method starts with no cost measures. In this case study there were no no-cost measures among those shortlisted in the auditing process though ( Figure 16). To maximize the benefits for the business owner the ring-fencing process began with the low-cost measures. The first ECM which was applied is the extraordinary maintenance of the PV-plant (restoration of the PV-strings). The main checks to perform during PV-plant maintenance are the verification of energy production, the inspection of breaker closing, the cleaning of glass dirt, the visual inspection of cable and enclosure, the maintenance of the sun-trackers’ motors ( Spertino & Corona, 2013). After that a second low-cost measure was selected, which is the optimization of the air distribution network of the compressed air system. The third measure installed was the control unit of the compressed air system. In fact, to have low annual operating costs compressors need to operate with efficient control modes and must not be oversized ( Kissock, 2005; Saidur *et al.*, 2010; Wright, 2008; Challenge, 2002). Moreover, an improved system management by means of installation of a new control unit will reduce the fluctuations in the air production, will establish a stable pressure, will improve the efficiency in the production of compressed air, will increase the overall stability of the system resulting in a better-balanced system ( Nehler *et al.*, 2018). The last ECM installed was a new control unit for the aspiration system. It has been recently shown that the recirculation of air flow through an aspiration system allows an energy consumption’s reduction of the ventilator even though that comes at the expense of reduction of the volume of the aspired air ( Kireev *et al.*, 2018).

Throughout the whole process of implementing energy efficiency measures in a small industrial business, human factors are of great importance to ensure a smooth implementation of the energy efficiency project. The decision making of the business-owner with respect to the implementation of energy efficiency measures to achieve the economic benefits of minimizing the life-cycle costs was likely affected by their sensitivity to the first costs of energy efficiency measures (efficient devices and equipment typically cost more than less efficient versions) ( Reddy, 2000). Another factor which can affect the decision of the business owner to continue pursuing the implementation of the energy efficiency plan is the fear of possible presence of hidden costs, such as those which can be associated to production disruptions, overheads, cost of collecting and analysing information ( Jaffe & Stavins, 1994; Rohdin & Thollander, 2006). The analysis of this case study revealed that the contractual form and the ring-fencing model used to deliver energy efficiency were liked by the client. The client did not respect the suggested order of interventions and decided to accept an increased pay-back time. Moreover, the client decided not to implement all the suggested EEMs in the list. However, the SPEEDIER expert’s consultancy fees were paid.

## Discussion

In the current state-of-the-art energy management, energy auditing and implementation of energy efficiency projects are based on the PDCA (Plan-Do-Check-Act) cycle (sometimes also known as the Deming Cycle) which enabled to achieve energy efficiency improvements and cleaner production in SMEs ( Prashar, 2017; Silva *et al.*, 2017). This methodology was developed back in 1930 when some exclusive products began to face the competition of new somewhat similar products and the quality management became one of the new market drivers ( Silva *et al.*, 2017). The application of the PDCA cycle to energy optimization in energy-intensive SMEs has been proposed to create a strategic approach to develop energy efficiency measures (EEMs) considering both technical and managerial dimensions, improving the traditional approaches which focused mostly on the technological improvements at the operational level ( Prashar, 2017). In the first step (Plan) the energy savings activities are identified, and an action plan is developed; this step may involve the creation of energy management roles and requires performing thorough energy audits. The second step (Do) concerns the implementation of the action plan and the preliminaries that require communication, awareness and motivation of the staff involved in the action plan. The third step (Check) is the periodic monitoring, analysis and reporting of the energy performances using key performance indicators (KPIs) previously defined. The fourth and last step (Act) involves conducting periodic management reviews and updates of the initial action plan to include new EEMs. It can be noticed how this approach does not consider in an explicit manner the budget constraints which are often the most significant barriers to energy efficiency in the SMEs and lacks the vision about a staggered implementation of the energy savings activities in the planning stage, which is the key concept introduced in this paper to enable the implementation of more EEMs using the energy savings previously accumulated and boost the energy efficiency in SMEs. In our research on energy efficiency in SMEs we found that the assumption that high-cost EEMs can be funded by the owner of the SME are too optimistic and that the sector of energy intensive SMEs needs the support of a financing mechanism for the implementation of medium to high-cost interventions. In this paper the ring-fencing mechanism has been proposed as a financing mechanism allowing the undertake of costly EEMs by means of the energy savings previously accumulated implementing no-cost and low-cost EEMs. The adoption of the ring-fencing methodology requires a deeper auditing and planning of the identified EEMs with respect to the plan step of the PDCA cycle to identify not only the efficiency measures which need to be prioritized, but a detailed multi-stage plan for their implementation considering budget constraints, cost of EEMs and annual savings produced by each energy efficiency measure. The benefit of a multi-stage model to allow a gradual integration of an energy management system in a company was identified in ( Javied *et al.*, 2015). However, the proposed approach was limited to only three stages, consisting respectively in the implementation of the basic package implementation (stage I), the enhanced package (stage II) and the sustainability package (stage III). Although the model proposed in ( Javied *et al.*, 2015) considered some elements of a multi-stage energy efficiency planning, there are assumption which are unnecessary and may affect the performances of the proposed approach in delivering a highly valued energy efficiency plan. In particular, the stage I considers the implementation of so-called quick wins which refers to the measures delivering benefits that a company can achieve rapidly, with minimal effort and low investment. The central aspect of delivering an enhanced package in stage II is the structure and process organisation and the clear definition of responsibilities, information, and communication channels. These organisational aspects (if not adequately addressed) may be the key potential barriers to an effective energy management in which EEMs can be continuously coordinated and implemented. The sustainability package considered in stage III is determined by another internal audit where a strategic and systematic optimization of energy consumption is performed. The results of the stage III audit are presented to the top-management and the company can apply for ISO 50001 certification if fulfilling all the requirements. It can be noticed that there is no upfront detailed multi-stage planning of the EEMs and that the multi-stage process appears to be a consequence of not having addressed simultaneously the aspects related to both organisational and technical dimensions of energy efficiency with a deeper energy audit in the planning stage of the energy efficiency process as it was performed in the case studies described in this paper. When the quick wins measures are implemented in stage I there is no detailed plan yet on how to continue with more costly and powerful measures. In stage I it is still unclear what would the measures of the enhanced package be and the organisational requirements to get them implemented in the company. In stage III another audit is required to check whether energy efficiency can be still optimised, if there are measures which were not considered in the previous audits and the management agrees to continue in the process. This approach obviously lacks the optimality of the ring-fencing methodology proposed in this paper, where all the EEMs are identified at the planning stage through a thorough energy audit and are then coordinated in a multi-stage implementation plan such that the rewards associated with the energy savings are maximised. Other auditing processes for resources and energy efficiency applicable to industrial or service companies focus only on the identification of a preliminary set of investment opportunities and their financial and technical assessment, lacking a vision for long-term, staggered implementation of EEMs ( Maffini *et al.*, 2021). In this paper we have reported the successful demonstration (three case studies) of a methodology for enhancing the energy auditing process and the planning of EEMs by means of a mechanism for multi-stage energy efficiency implementation which enable to finance advanced EEMs from the energy savings obtained installing no-cost and low-cost measures. This methodology enhances the state-of-the-art of energy auditing and energy efficiency planning identifying and unlocking many more opportunities for energy efficiency improvements in energy intensive SMEs. 

## Conclusions

This paper has presented a novel energy efficiency auditing process for SMEs including a ring-fencing mechanism for multi-stage planning of EEMs, which enables to finance the installation of new measures using the energy savings delivered by the EEMs previously implemented, which are accumulated until the end of the energy efficiency project. The ring-fencing model has been validated on three case studies which were part of the pilot demonstrations developed in the H2020 project SPEEDIER. The case studies show the effectiveness of the ring-fencing model in driving the selection of the most appropriate EEMs throughout multi-annual retrofitting projects. The auditing process delivered has been successful in convincing the decision-makers that a planning over multiple periods of EEMs’ installation is advantageous to overcome the barriers related to the limited access to capital, with respect to the case where the selection of the technologies and the investment on them is performed at once for the considered planning period. Furthermore, the auditing process substantiates the finding that decision making for energy efficiency in SMEs shows a complex decision structure and that decision-makers may not be able to optimise their choices because of lack of time, attention, and the ability to adequately process information. The ring-fencing method investigated in this paper is a tool to help overcoming bounded rationality issues (resulting in the use of sub-optimal routines or rules of thumb) and improving opportunities for achieving the selection of the right EEMs among all those available, such that financial benefits are maximized (as well as environmental benefits if agreed during the audit).

## Data availability

The case study data is available as public deliverable of the project, which can be accessed via SPEEDIER website D9.4.

## Ethics and consent

Ethical approval and consent were not required.
