# Supplementary material for: The Ring Fencing Mechanism: a case study of innovative self-financing approach for energy efficiency upgrades
Source: Open Res Eur. 2022 May 5;2:53. [Version 1] doi: 10.12688/openreseurope.14731.1 (PMC10445892; doi:10.12688/openreseurope.14731.1)
Supplement: Peer review history

# Peer review: Reviewer response for version 1

**DOI:** 10.21956/openreseurope.15913.r29664

**Published:** 2022-08-25

**Authors:** Ivan Garcia Kerdan

**Affiliations:** 1 School of Engineering and Sciences, Tecnologico de Monterrey, Mexico City, Mexico

The manuscript investigates the impact of implementing a ring fencing mechanism approach to overcome financial barriers that companies face when implementing energy saving measures. The study's main contribution is to understand the implications of such methods in real case studies. 

 In current research practice, several studies already exist that investigate the implications of financing in energy saving projects. Overall, the study is well written, clear and tackles an important problem of financing mid to long-term projects. However, the only concern is the lack of clarity on how the energy savings have been quantified. 

 The paper could be improved if the previous concern is answered; thus, minor technical rework is necessary before it can be considered for indexing.

 Although most of the literature is appropriate, the introduction section could show cases when typical financing methods are implemented. This will provide more information about the limitations of such mechanisms, especially when applied to SMEs. Despite the work carried out, originality needs to be further explained or enhanced.

 The results section shows interesting results considering the chosen methodology. The section provides a concise discussion section reflecting a good understanding of the modelling outputs. The authors provide a critical reflection of the three case studies by providing insight for policy and decision-makers under the current economic environment.

Where applicable, are recommendations and next steps explained clearly for others to follow? (Please consider whether others in the research community would be able to implement guidelines or recommendations and/or constructively engage in the debate)

Yes

Does the article adequately reference differing views and opinions?

Partly

Are all factual statements correct, and are statements and arguments made adequately supported by citations?

Yes

Is the rationale for the Open Letter provided in sufficient detail? (Please consider whether existing challenges in the field are outlined clearly and whether the purpose of the letter is explained)

Yes

Is the Open Letter written in accessible language? (Please consider whether all subject-specific terms, concepts and abbreviations are explained)

Yes

Reviewer Expertise:

Energy modelling, energy efficiency building sector

I confirm that I have read this submission and believe that I have an appropriate level of expertise to confirm that it is of an acceptable scientific standard.

# Peer review: Reviewer response for version 1

**DOI:** 10.21956/openreseurope.15913.r29661

**Published:** 2022-08-05

**Authors:** Alberto Cerezo-Narváez

**Affiliations:** 1 Departamento de Ingeniería Mecánica y Diseño Industrial, Escuela Superior de Ingeniería, Universidad de Cádiz, Puerto Real, Spain

The authors propose a methodology to improve the implementation of energy efficiency measures in SMEs that perform energy audits. However, in order to be finally indexed, the paper should attend some questions related below.

 Abstract:

 The barrier of lack of capital for the implementation of energy efficiency measures can extendable to private developers, resident associations, small public administrations, etc. Accordingly, if only SMEs are referred, the explanation to this constraint should be justified or explained. In addition, the proposed ring-fencing approach should be better summarized. It can be noted that some measures may be very beneficial economically but not environmentally (good but not so good). And vice versa. Therefore, economic and environmental aspects should initially be treated separately.

 Introduction:

 References to justify the low participation of SMEs in energy audits should be updated, as the authors should ensure that this trend remains low 12 years later. The gap (to be solved) should be better highlighted. For instance, cost-optimal energy retrofit proposal provided by auditors could increase the rate measures proposed vs measures implemented. The quality of the Figure 1 should be improved because the factors are not ordered in the same way in all countries and some percentages are not clear. Many references date back 10, 11 or 12 years. In these years property owners and occupiers may have changed their position due to the energy context (fuel prices, electricity prices, supply shortages, etc.). And in the last 2 years, even more so. Energy efficiency projects reduce the amount of consumed energy but also make the consumption not eliminated come (partly) from renewable sources.

 ISO 50001 should be taken into account since it was conceived to provide a framework for more efficient use of energy.

 Ring Fencing Mechanism:

 The paper does not analyze the compatibility of these sequential proposals. This way of ordering interventions discriminates (or may discriminate) against those options that require larger investments to begin with. Are all larger investments ruled out? Larger by how much? Some measures are independent, but others are linked to previous decisions, and are made with future decisions in mind. While the idea sounds good, these drawbacks should be addressed.

 Methodology:

 This section should be joined to the previous one. The use of case studies as a methodology is widely used in the literature, but in this paper, the authors have not sufficiently justified and discussed for it. How they are chosen, how many are chosen, and what their characteristics should be (representativeness, uniqueness, etc.).

 Case studies: Decision making about measures selection should be provided. This enhances the testing of the usefulness of the methodology. There are no alternatives to be followed. The methodology does not help to analyze which measures are independent and which ones are dependent. In the second case, alternatives should be raised. Time-dependent graphs should be presented that help to see how savings from the first measures can be used as available capital to undertake the next measures, predicting (through simulations) how much additional capital (if required) is needed to undertake each of the measures chosen from the proposed measures. In this regard, information provided in Figure 16 (for instance) could be a good initial point.

 Many data from this section could be laid out as supplementary material because its length is not proportional with the rest of the paper.

 Discussion:

 Cost-optimal and financial-optimal approaches are not considered to be compared with the chosen one (the proposed by authors). I mean, Is the best choice to begin always by the cheapest?

 Different sequences may be proposed, each of one can provide different savings distributed along years, helping or hindering future investments. The final savings through the remaining lifespan of the building should also be taken into account.

 Conclusion:

 Which are the improvements into the 3 case studies? The energy efficiency has improved, but no specific nor measurable findings are included at the end. Limitations are not considered. Future research is not suggested.

Where applicable, are recommendations and next steps explained clearly for others to follow? (Please consider whether others in the research community would be able to implement guidelines or recommendations and/or constructively engage in the debate)

Partly

Does the article adequately reference differing views and opinions?

Partly

Are all factual statements correct, and are statements and arguments made adequately supported by citations?

Partly

Is the rationale for the Open Letter provided in sufficient detail? (Please consider whether existing challenges in the field are outlined clearly and whether the purpose of the letter is explained)

Partly

Is the Open Letter written in accessible language? (Please consider whether all subject-specific terms, concepts and abbreviations are explained)

Yes

Reviewer Expertise:

Energy Efficiency in Building, Building Technology, Sustainable Architecture, Green Building, Bioclimatic Architecture

I confirm that I have read this submission and believe that I have an appropriate level of expertise to confirm that it is of an acceptable scientific standard, however I have significant reservations, as outlined above.

# Peer review: Reviewer response for version 1

**DOI:** 10.21956/openreseurope.15913.r29287

**Published:** 2022-05-23

**Authors:** Tony Day

**Affiliations:** 1 Independent Energy Research Consultant, Cambridge, UK

The article covers an important topic of how to encourage SMEs to invest in energy savings measures. It presents research to show the barriers that exist to this, and proposes a methodology that can provide a staged approach to energy management investment. The financial savings that accrue from low and no-cost measures can be used to pay for future higher cost measures. What is missing is an explanation of how such savings can be ring-fenced in practice. This may be a purely accounting exercise, or might involved money transfers to identified holding accounts. SMEs will have different methods for dealing with capital investments, and it is important that the accrued savings are clearly marked for future energy efficiency investments if this is to be successful. It would be helpful if this article could outline how this ring-fencing occurs.

 The other missing piece is how the measurement and verification process should be implemented. This can be relatively expensive and is not a trivial exercise. The article should indicate which party is responsible for conducting this, and what level of costs are likely to be involved.

 Without these additions, any practitioner would have to decide on this approach for themselves. International Standards such as ISO 50015 would be important to mention in respect of the latter point.

 In a number of places, the language is difficult to follow. Overall the message is clear, but some arguments are a little difficult to follow. A thorough edit is advised.

Where applicable, are recommendations and next steps explained clearly for others to follow? (Please consider whether others in the research community would be able to implement guidelines or recommendations and/or constructively engage in the debate)

Partly

Does the article adequately reference differing views and opinions?

Yes

Are all factual statements correct, and are statements and arguments made adequately supported by citations?

Yes

Is the rationale for the Open Letter provided in sufficient detail? (Please consider whether existing challenges in the field are outlined clearly and whether the purpose of the letter is explained)

Yes

Is the Open Letter written in accessible language? (Please consider whether all subject-specific terms, concepts and abbreviations are explained)

Partly

Reviewer Expertise:

Energy and carbon management and the application of renewable energy in buildings and the built environment. Energy monitoring and targeting, and measurement and verification. Optimisation of mechanical and electrical building services systems.

I confirm that I have read this submission and believe that I have an appropriate level of expertise to confirm that it is of an acceptable scientific standard, however I have significant reservations, as outlined above.
